# One-dimensional carbon nanotube@barium titanate@polyaniline multiheterostructures for microwave absorbing application

**DOI:** 10.1186/s11671-015-0875-6

**Published:** 2015-04-11

**Authors:** Qing-Qing Ni, Yao-Feng Zhu, Lu-Jun Yu, Ya-Qin Fu

**Affiliations:** Key Laboratory of Advanced Textile Materials and Manufacturing Technology of the Ministry of Education, Zhejiang Sci-Tech University, No.928 Second Avenue Xiasha Higher Education Zone, Hangzhou, 310018 People’s Republic of China; National Engineering Lab for Textile Fiber Materials and Processing Technology, Zhejiang Sci-Tech University, No.928, Second Avenue, Xiasha Higher Education Zone, Hangzhou, 310018 People’s Republic of China

**Keywords:** BaTiO_3_, Polyaniline, Carbon nanotube, Heterostructure, Microwave absorption

## Abstract

Multiple-phase nanocomposites filled with carbon nanotubes (CNTs) have been developed for their significant potential in microwave attenuation. The introduction of other phases onto the CNTs to achieve CNT-based heterostructures has been proposed to obtain absorbing materials with enhanced microwave absorption properties and broadband frequency due to their different loss mechanisms. The existence of polyaniline (PANI) as a coating with controllable electrical conductivity can lead to well-matched impedance. In this work, a one-dimensional CNT@BaTiO_3_@PANI heterostructure composite was fabricated. The fabrication processes involved coating of an acid-modified CNT with BaTiO_3_ (CNT@BaTiO_3_) through a sol–gel technique followed by combustion and the formation of CNT@BaTiO_3_@PANI nanohybrids by *in situ* polymerization of an aniline monomer in the presence of CNT@BaTiO_3_, using ammonium persulfate as an oxidant and HCl as a dopant. The as-synthesized CNT@BaTiO_3_@PANI composites with heterostructures were confirmed by various morphological and structural characterization techniques, as well as conductivity and microwave absorption properties. The measured electromagnetic parameters showed that the CNT@BaTiO_3_@PANI composites exhibited excellent microwave absorption properties. The minimum reflection loss of the CNT@BaTiO_3_@PANI composites with 20 wt % loadings in paraffin wax reached −28.9 dB (approximately 99.87% absorption) at 10.7 GHz with a thickness of 3 mm, and a frequency bandwidth less than −20 dB was achieved from 10 to 15 GHz. This work demonstrated that the CNT@BaTiO_3_@PANI heterostructure composite can be potentially useful in electromagnetic stealth materials, sensors, and electronic devices.

## Background

Serious electromagnetic interference (EMI) pollution arising from the drastic development of telecommunication and gigahertz (GHz) electronic systems has aroused great interest in electromagnetic-absorber technology to solve the problem [1-5]. An ideal electromagnetic wave absorbing material should exhibit light weight, be thin, and have a strong wave absorption and wide frequency range response [6,7].

Carbon materials, with dielectric loss features, have been used as a very important component in microwave absorbers [8-10]. Brosseau et al. [9,11,12] investigated the dielectric properties of a series of carbon black-filled polymers, and the complex effective permittivity for carbon black-filled polymeric systems was well discussed. It is well known that neat carbon materials possess a very high conductivity, resulting in mismatch impedance, thereby inducing very limited microwave absorption [13,14]. Therefore, carbon nanotubes (CNTs) have been used as filler to prepare CNT/polymer composite microwave absorbers [15]. Recently, heterostructure-based CNT electromagnetic (EM) absorbers with good impedance matching have been fabricated, indicating that it could be an effective way to optimize the EM parameters and enhance the absorption performances [16,17]. Song et al. [18] fabricated ZnO-coated CNTs and dramatically improved their microwave absorption. Wang et al. [14] synthesized magnetite-decorated CNTs, which exhibited considerable EM absorbing ability. This was due to synergetic interactions between the magnetic nanocrystals and the CNTs. It has been observed that the interfaces of the composite materials also play an important role in EM absorption [19].

In order to meet further requirements of broadband microwave absorbers, multiple-phase heterostructure materials with new or enhanced EM absorption properties have been developed due to the interfacial polarization and confinement effect [13,20-22]. Polyaniline (PANI) is one of the most important conducting polymers with excellent environmental stability and tunable conductivity, and it has been considered as an ideal matrix or as a second phase incorporated with inorganic nanomaterials to achieve unique EM wave absorption [7,23-26]. However, the formation of multiple-phase composites with PANI encapsulated on the side surface of one-dimensional inorganic nanocomposites has been rarely reported.

The effect of absorbing materials is not only a strong absorption but also a wide absorption bandwidth that can be obtained by integrating the advantages of CNT, BaTiO_3_, and PANI with different loss mechanisms. PANI encapsulated on the surface of the composite may lead to an increase in matching impedance. In this report, we have coupled a sol–gel method and an *in situ* polymerization to successfully prepare a one-dimensional CNT@BaTiO_3_@PANI multiphase heterostructure composite. The structures, morphology, and conductive properties of the composite were fully characterized. Because of their special structural characteristics, well-matched characteristic impedances, and interfacial polarization induced by multiple interfaces in the composites, the multiple-phase heterostructure composites exhibit excellent EM absorption performances. Thus, one-dimensional CNT@BaTiO_3_@PANI heterostructures are very promising as a lightweight EM absorbing material.

## Methods

Multiwalled CNTs (MWCNTs) with diameter ranging from 40 to 70 nm were obtained from Wako Pure Chemical Reagent Co., Ltd., Chuo-ku, Japan. Oxidation of MWCNTs was carried out in hot, concentrated nitric acid. Barium acetate (Ba(CH_3_COO)_2_), tetraisopropyl titanate (Ti(OC_3_H_7_)_4_), acetic acid, hydrochloric acid, ammonium peroxodisulfate (APS), sodium dodecyl benzene sulfonate (SDBS), and ethanol were supplied by Wuxi Zhanwang Chemical Reagent Co., Ltd., Yixing, China. Aniline (99%) was supplied by Sinopharm Chemical Reagent Co., Ltd., Shanghai, China. All chemicals were used without further purification. Deionized water was used in all experiments.

### Preparation of oxidized CNTs

Typically, MWCNTs (approximately 0.5 g) were acidified by concentrated nitric acid with vigorous stirring at 115°C for 6 h, to obtain oxidized CNTs with a large number of oxygen-containing reactive groups on the ends and sidewall. The acid-oxidized CNTs were then collected by filtration and washed with deionized water until a neutral pH value was obtained in the washing solution.

### Synthesis of the BaTiO_3_ precursor

The BaTiO_3_ precursor was produced via a sol–gel method. The Ba and Ti cations were controlled at a 1:1 molar ratio. In a typical process, barium acetate (5 mmol) was dissolved into a mixture of acetic acid (5 mL) and anhydrous ethanol (20 mL) with vigorous stirring at 60°C water bath for 30 min, designated as solution A. Titanium isopropoxide (5 mmol) was dissolved into anhydrous ethanol (10 mL) containing deionized water (1 mL) with stirring for 15 min to make it homogeneous. That solution was then added to the mixed solution A slowly under continuous stirring conditions and maintaining a temperature of 60°C for 2 h and then aged at room temperature for 24 h.

### CNT@BaTiO_3_ heterostructures

In a typical fabrication experiment, the as-treated MWCNTs (100 mg) were dispersed in BaTiO_3_ sol solution by ultrasonication for 30 min, and then vigorous stirring was applied to the mixture suspension for reaction at 40°C for 4 h. The resulting suspension was filtrated, and the powder was dried and then calcined in a tube furnace at 700°C for 2 h under high-purity Ar atmosphere. CNT@BaTiO_3_ heterostructures were obtained.

### CNT@BaTiO_3_@PANI heterostructures

For fabrication of CNT@BaTiO_3_@PANI, a portion of the as-prepared CNT@BaTiO_3_ heterostructures (50 mg) were mixed with SDBS (20 mg) dispersed in 28 mL deionized water and then ultrasonicated for 2 h to obtain a uniform suspension. HCl (5 mL, 1 mol/L) and aniline (5 mmol) were subsequently added under stirring condition. Until homogeneous suspension was achieved, APS aqueous solution (5 mmol of APS in 10 mL of deionized water) was dropwise added to the suspension. The polymerization process was applied in an ice bath for 24 h under stirring. The resulting precipitations were washed with deionized water and ethanol, followed by drying in an oven (50°C).

### Characterization

The morphology and microstructure of the products were characterized using field emission scanning electron microscopy (FE-SEM; Hitachi S-4800, Hitachi, Ltd., Chiyoda-ku, Japan) and transmission electron microscopy (TEM; JEOL JEM-2100 F, JEOL Ltd., Akishima-shi, Japan) with an accelerating voltage of 200 kV. The crystal structure of the prepared powders was analyzed with an X-ray diffractometer (D8-Discover, Bruker AXS, Billerica, MA, USA), using Cu K*α* radiation. Fourier transform infrared spectroscopy (FT-IR) was performed using a Nicolet 5700 FTIR spectrometer (Thermo Electron Corp, Waltham, MA, USA) with KBr pellets. Thermogravimetric analysis (TGA) was carried out in nitrogen atmosphere from room temperature to 700°C at a heating rate of 5°C/min using a SDTA851e analyzer (Mettler-Toledo, Greifensee, Switzerland).

The composite samples used for electromagnetic measurements were prepared by loading the products in paraffin wax. The powder-wax compound was then pressed into toroidally shaped samples (*φ*_out_ = 7 mm, *φ*_inner_ = 3 mm, *H*_thickness_ = 2 mm) for complex relative permittivity *ε* (*ε = ε′* − *jε*″) and magnetic permeability *μ* (*μ = μ′* − *jμ*″) measurements with a vector network analyzer (37247D, Anritsu Co., Ltd., Atsugi-shi, Japan) in the 0.5 to 15 GHz range.

## Results and discussion

### Morphology and structure analysis

**Figure 1:**
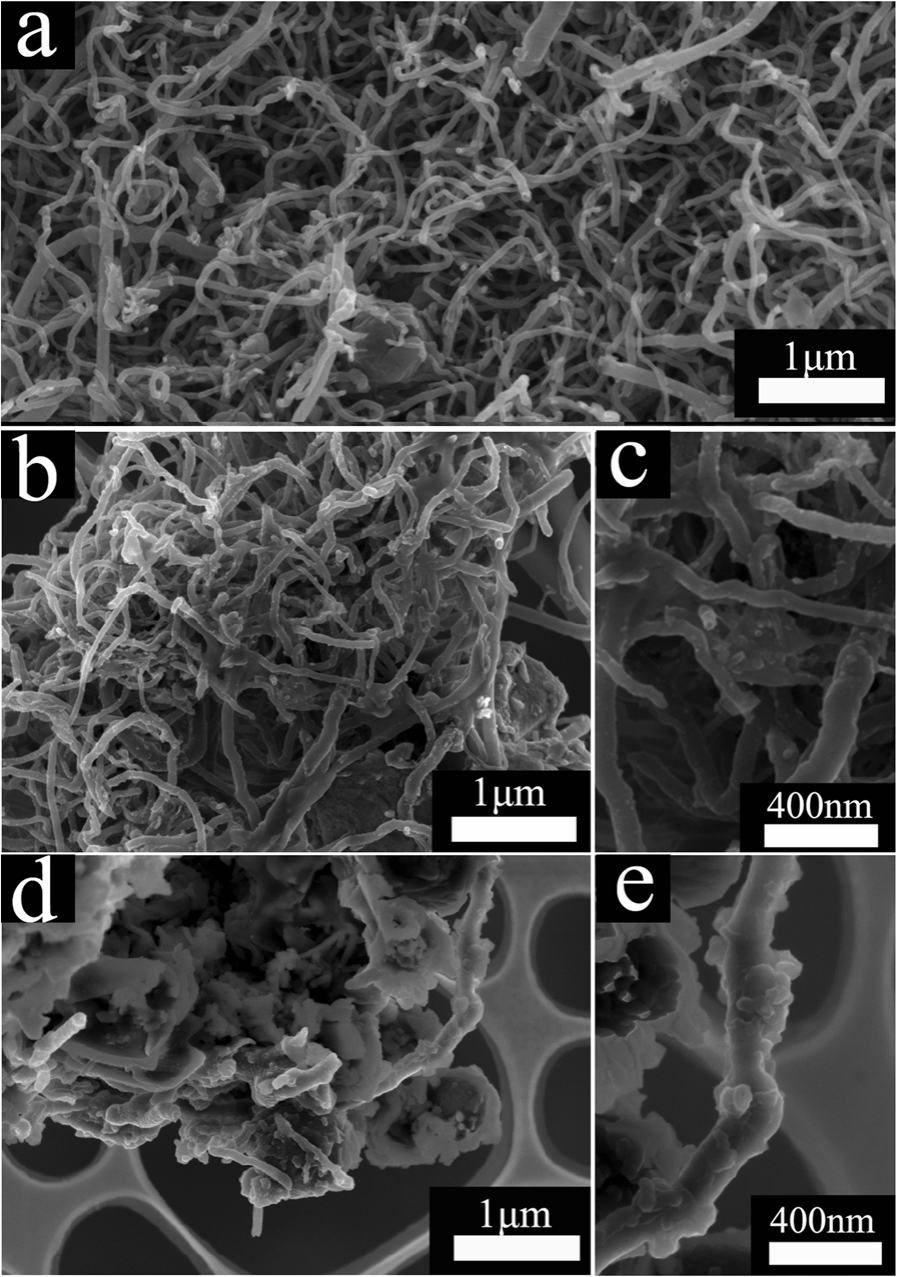
Representative FE-SEM images of (**a**) CNTs, (**b**, **c**) CNT@BaTiO_3_ at different resolutions, and (**d**, **e**) CNT@BaTiO_3_@PANI at different resolutions.

Representative FE-SEM images for CNTs, CNT@BaTiO_3_, and CNT@BaTiO_3_@PANI composites are shown in Figure 1a,b,c,d,e. The neat CNT morphology is displayed in Figure 1a as endless, disentangled, smooth surfaces, and the diameter of each nanotube was about 40 to 70 nm. For the CNT@BaTiO_3_ composites, seen in Figure 1b,c, it can be clearly observed that BaTiO_3_ adhered onto the surface of the CNT, and the surface was no longer smooth. The morphology of the CNT@BaTiO_3_@PANI composites is shown in Figure 1d,e. The CNT@BaTiO_3_@PANI composites exhibit hierarchical structures and the average diameter increased after polymerization, assuring that the PANI was well coated on the surface of CNT@BaTiO_3._

**Figure 2:**
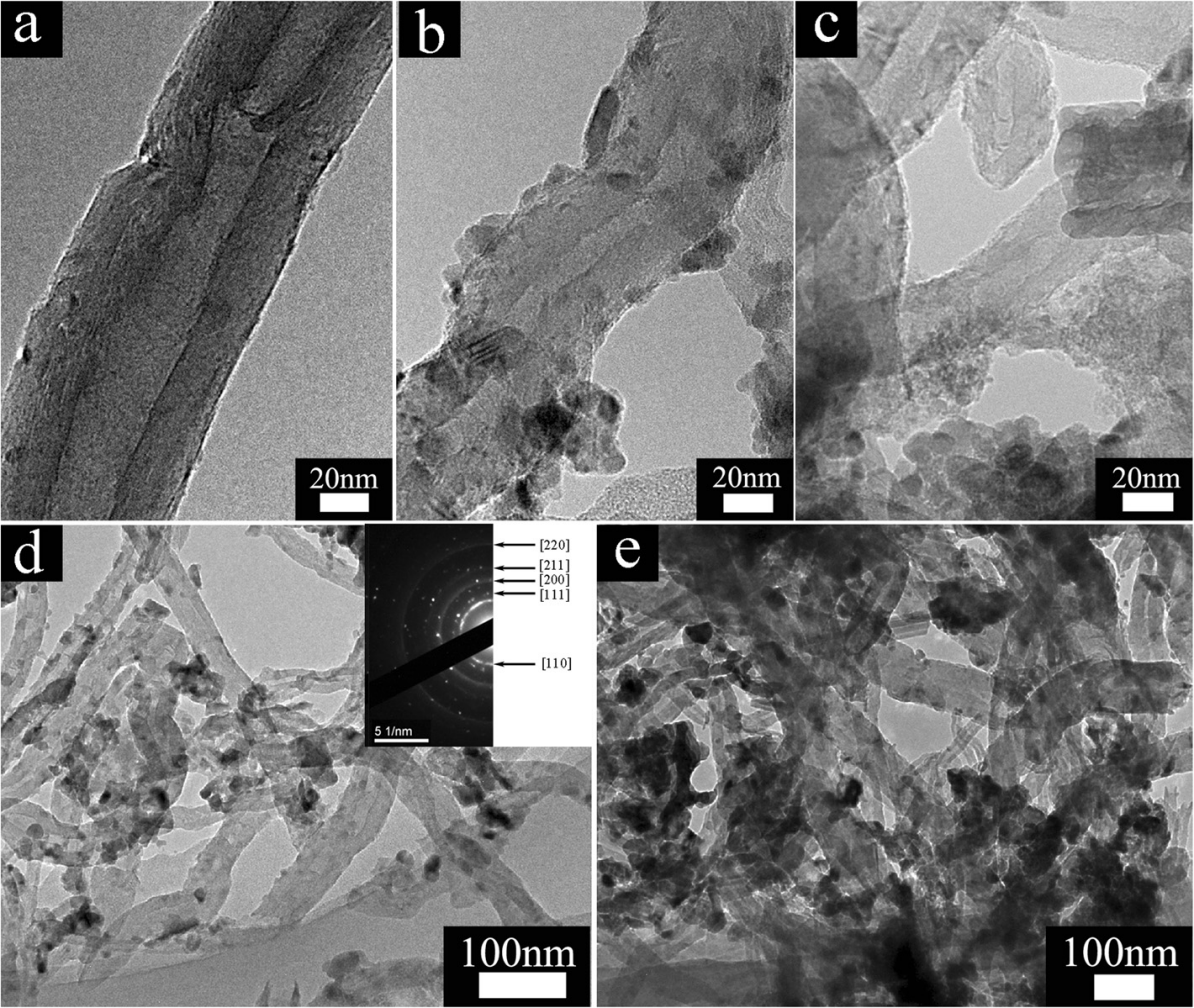
TEM images of (**a**) CNTs, (**b**, **d**) CNT@BaTiO_3_ at different resolutions, and (**c**, **e**) CNT@BaTiO_3_@PANI at different resolutions. The inset in (**d**) is the corresponding electron diffraction pattern.

The morphology and structure of the CNT, CNT@BaTiO_3_ composites, and CNT@BaTiO_3_@PANI composites were further analyzed by TEM. Figure 2b, d shows the TEM images of the CNT@BaTiO_3_ composites at different resolutions. It was revealed that the BaTiO_3_ films are well coated on the CNT surface as expected. The top right inset in Figure 2d shows the selected-area electron diffraction (SAED) pattern for the CNT@BaTiO_3_ composites. The diffraction rings in the pattern corresponded to the (110), (111), (200), (211), and (220) crystalline planes of BaTiO_3_; this was further confirmed by X-ray diffraction (XRD). Figure 2c,e exhibits the high- and low-magnification TEM images of CNT@BaTiO_3_@PANI composites, which clearly indicated that the polyaniline macromolecules encapsulated on the surface of the CNT@BaTiO_3_ composites. The layer of coated polyaniline had a mean wall thickness of approximately 15 nm, as measured from TEM images, which agreed with the FE-SEM observation results obtained from Figure 1. It can be observed that CNT@BaTiO_3_@PANI composites with a typical multiphase heterostructure were successfully fabricated.

**Figure 3:**
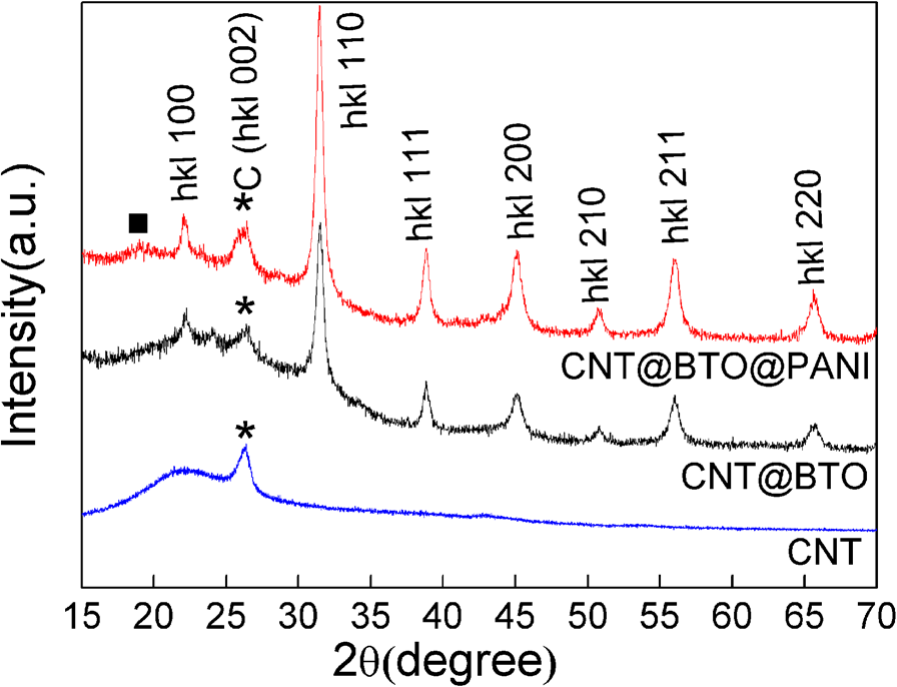
XRD pattern of the CNTs, CNT@BaTiO_3_ composite, and CNT@BaTiO_3_@PANI composite. Black square, diffraction peak of PANI; asterisks, diffraction peaks of CNTs.

The crystalline phase and phase composition of the bare CNTs and the composites were analyzed by XRD, as shown in Figure 3. The diffraction peak centered at approximately 2*θ* = 26.2°, corresponding to the (002) reflections of highly graphitized arc-discharge CNTs, and a broad diffraction peak appearing at approximately 2*θ* = 22.2° originated from the (amorphous carbon) disordered graphite structure [27,28], which indicated that the CNTs were well preserved during the synthesis of the heterostructures. Meanwhile, for the CNT@BaTiO_3_@PANI composites, the characteristic diffraction peak at approximately 2*θ* = 19.8° was observed, corresponding to the amorphous PANI peak [3]. In addition to the characteristic reflections from CNTs and PANI, all of the diffraction peaks unaccounted for by the characteristic of the CNTs and PANI could be indexed to the cubic crystal structure of BaTiO_3_, which matched well with the reported data (JCPDS No. 31–0174); no meaningful change was observed before and after the PANI polymerization. It can be concluded that the CNT@BaTiO_3_@PANI heterostructure composites were formed. It was noted that the weak peak strength of the polyaniline in the composites was due to its low crystallinity.

**Figure 4:**
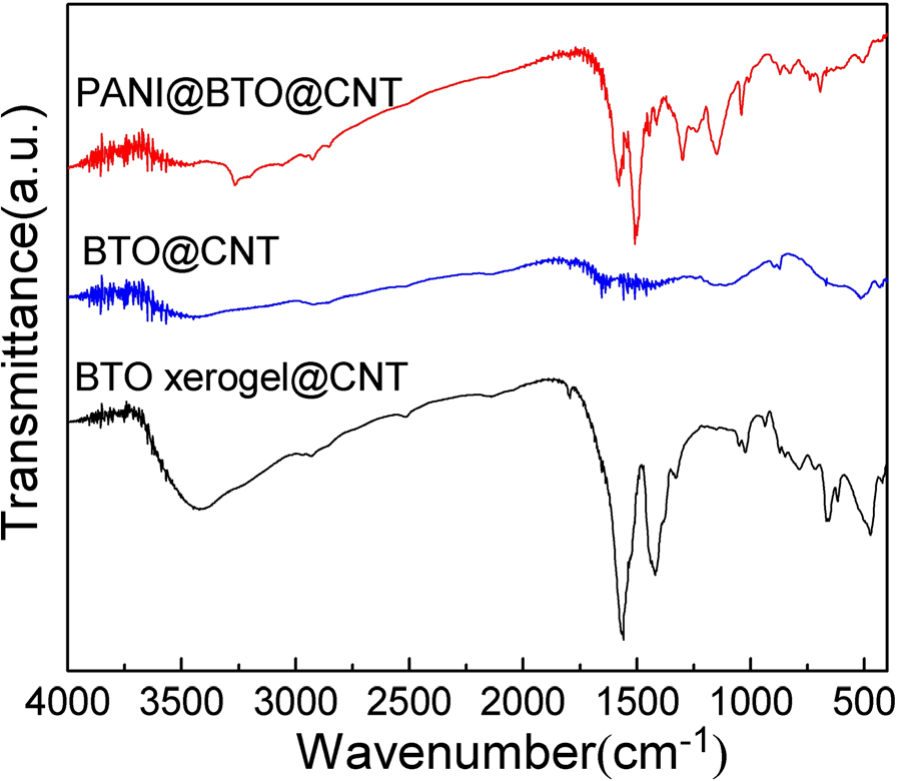
FT-IR spectra of the CNT@BaTiO_3_ xerogel, CNT@BaTiO_3_ composite, and CNT@BaTiO_3_@PANI composite.

The formation of samples was further evaluated by FT-IR measurement, as shown in Figure 4. The FT-IR spectrum of CNT@BaTiO_3_ xerogel shows the typical strong absorption peaks of the acetate ligand chelating the titanium compound at around 1,420 and 1,560 cm^−1^, respectively. These two absorption bands are caused by symmetric and asymmetric vibrations of the acetate group [29]. The absorption bands centered at 666 and 491 cm^−1^ can be attributed to Ti-O stretching vibrations and are characteristic of BaTiO_3_ [26]. These absorption bands almost completely disappeared from the spectrum after the CNT@BaTiO_3_ xerogel was calcined in a tube furnace, except for the absorption peak at around 500 cm^−1^ which is characteristic of BaTiO_3_. The FT-IR spectrum of the CNT@BaTiO_3_@PANI composites shows main absorption bands situated at 1,593, 1,513, 1,297, 1,235, 1,144, 827, and 505 cm^−1^. The peaks at 1,593 and 1,513 cm^−1^ correspond to the stretching vibration of C = N in the quinine ring and the C = C in the benzene ring, respectively [26,28]. The peak at 1,297 cm^−1^ for the heterostructures is due to the C-N (C_aromatic_-N) stretching vibration in PANI. The characteristic band at 1,144 cm^−1^ can be attributed to the benzene-NH^+^ vibration [30,31]. The peaks between 900 and 700 cm^−1^ correspond to the C-H bending absorption for the benzenoid unit of PANI [28]. And the absorption band at 1,235 cm^−1^ (C-N^+^ stretching vibration) indicates the doped form of PANI. The characteristic band for BaTiO_3_ at around 505 cm^−1^ was also observed. These findings agree very well with the XRD results.

**Figure 5:**
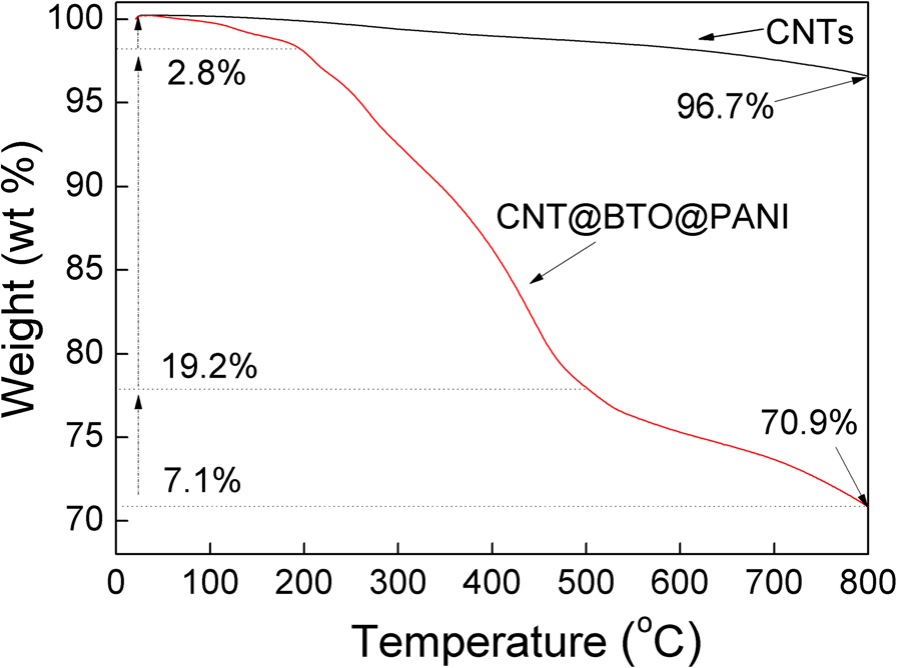
Thermogravimetric analysis of the CNT@BaTiO_3_@PANI composite.

Thermal stability is an important property of the CNT@BaTiO_3_@PANI composites. The TGA weight loss curve shown in Figure 5 demonstrates the thermal stability of the CNT@BaTiO_3_@PANI composites, and it also shows the mass fraction of PANI coated on the surface of the composites. For the CNTs, it was found that the total weight loss of the CNTs was approximately 3.3%, and was mainly related to the thermal degradation of the carboxyl and hydroxyl groups formed on the surface of the CNTs as well as some loss of surface absorbed water at low temperature [32]. For the CNT@BaTiO_3_@PANI composites, it can be seen that three weight loss steps take place over the scanning temperature range from 25°C to 700°C. The first weight loss step (approximately 2.8%), from 25°C to approximately 200°C, corresponds to the loss of surface absorbed water and bound water molecules in the sample. The second weight loss step (approximately 19.2%), between approximately 200°C and 500°C, is caused by the decomposition of the low molecular weight oligomer and the skeleton of polyaniline [33,34]. The steepest weight loss observed (approximately 7.1%) may be caused by the decomposition of the residues formed in the degradation process of polyaniline.

### Conductivity of the CNT@BaTiO_3_@PANI composites

The sample was pressed into circular sheets (thickness = 0.5 mm, diameter = 1 cm). The values of conductivity by a four-probe measurement for the CNT@BaTiO_3_ composites and CNT@BaTiO_3_@PANI composites were 0.39 and 1.51 S/cm, respectively. For the as-prepared CNT@BaTiO_3_ composites, the conductivity deceases significantly compared to the high conductivity of the raw CNTs. This was mainly caused by the insulating behavior of the BaTiO_3_ coating on the surface of the CNTs which hinders the charge transfer. Notably, the CNT@BaTiO_3_@PANI composites showed an evident increase in conductivity compared with the CNT@BaTiO_3_ composites. It can be attributed intrinsically to the conducting polymer due to the presence of a conjugated *π* electron system in their structure. The conductivity of the composites was in the magnitude of 10^−1^ to 10^0^ S/cm, which can be considered as an appropriate conductivity level for a material used as a microwave absorber.

### Electromagnetic wave absorption properties of the CNT@BaTiO_3_@PANI composites

**Figure 6:**
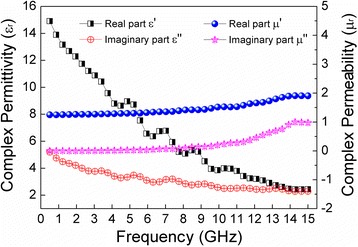
Complex permittivity and permeability of the CNT@BaTiO_3_@PANI composite. With 20 wt % loadings in paraffin wax of 2-mm thickness in the frequency range of 0.5 to 15 GHz.

It is well known that the microwave absorption properties of an absorber are highly associated with its complex relative permittivity and complex magnetic permeability, where the real parts represent the storage of electric and magnetic energies, whereas the imaginary parts represent the loss of both energies [26]. We independently measured the complex relative permittivity and the magnetic permeability of the CNT@BaTiO_3_@PANI to get a better understanding of its microwave absorption properties. Figure 6 shows the real and imaginary parts of the complex relative permittivity (*ε′*, *ε*″) and magnetic permeability (*μ′*, *μ*″) measured for the CNT@BaTiO_3_@PANI, with 20 wt % loadings in paraffin wax with a thickness of 2 mm. As shown in Figure 6, it can be seen that the real parts of the complex relative permittivity (*ε′*) of the CNT@BaTiO_3_@PANI-filled sample was markedly decreased with an increasing frequency, which can be attributed to the fact that the dipoles present in the material find it increasingly difficult to maintain the phase orientation with the electric vector of the incident radiation [35]. Samples that have a relatively high dipole density cannot reorient themselves along with the applied electric field, which may be responsible for the observed rapid decrease in *ε′* of the CNT@BaTiO_3_@PANI-filled sample. Meanwhile, the imaginary part of the complex relative permittivity (*ε*″) of the CNT@BaTiO_3_@PANI-filled sample was found to steadily decrease with an increase in frequency in the investigated region. Notably, both real (*ε′*) and imaginary (*ε*″) relative permittivity of the composites show several small resonance peaks near 4.0, 6.0, and 8.0 GHz, which may be mainly associated with the interface in the multiphase heterostructure of the CNT@BaTiO_3_@PANI composites [34,36]. The real and imaginary parts of the complex permeability of the CNT@BaTiO_3_@PANI composites are also shown in Figure 6. It can be observed clearly that the real part *μ′* of the CNT@BaTiO_3_@PANI-filled sample remains almost constant in the low frequency range and then slowly increases with the increase of the frequency. Interestingly, the imaginary part *μ*″ of the CNT@BaTiO_3_@PANI filled-sample behaved similarly with respect to the frequency as well as the real part *μ′*. The relatively low conductivity of the CNT@BaTiO_3_@PANI composites may lead to a decrease in the number of eddy currents in the system induced by the electromagnetic waves [37]. It can also be seen that the *μ*″ values of the sample have a broad resonance peak near 14.2 GHz, which may be associated with the local confinement, the natural resonance, and the exchange resonance loss [38,39].

To further prove the dependence of the microwave absorption properties on the complex relative permittivity and the magnetic permeability, the reflection loss (RL) properties of the samples were calculated according to the transmission line theory, as follows:

1 $$ \mathrm{R}\mathrm{L}=20\; \log \left|\frac{{\mathrm{Z}}_{\mathrm{in}}-1}{{\mathrm{Z}}_{\mathrm{in}}+1}\right| $$

**Figure 7:**
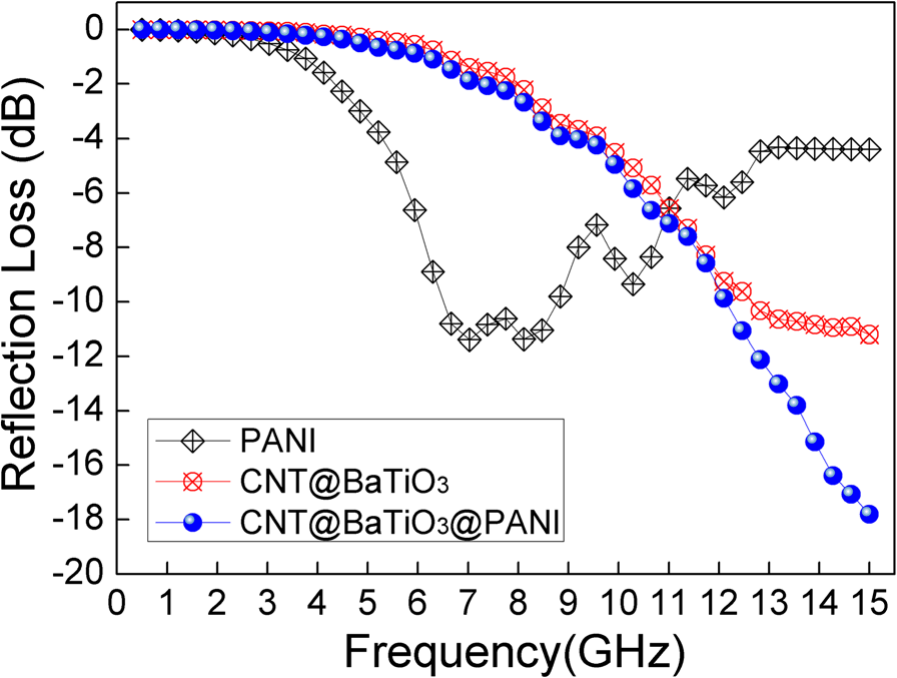
Reflection loss curves for PANI, CNT@BaTiO_3_, and CNT@BaTiO_3_@PANI composite. With 20 wt % loadings in paraffin wax of 2-mm thickness in the frequency range of 0.5 to 15 GHz.

The normalized input impedance (*Z*_in_) is given by the formula:

2 $$ {\mathrm{Z}}_{\mathrm{in}}=\sqrt{\frac{\mu }{\varepsilon }} \tanh \left[j\left(\frac{2\pi fd}{c}\right)\sqrt{\mu \varepsilon}\right] $$

where *ε = ε′ − jε*″, *μ = μ′ − jμ*″, *f* is the microwave frequency in Hz, *d* is the thickness of the absorber in m, and *c* is the velocity of light in free space in m/s. Based on the electromagnetic parameters (the complex values of the relative permittivity and the magnetic permeability), the RL could be calculated for the given frequency with various thicknesses according to Equations 1 and 2. Figure 7 shows the calculated RL results of the PANI, CNT@BaTiO_3_, and CNT@BaTiO_3_@PANI with 20 wt % loadings in paraffin wax for the thickness of 2 mm. It can be seen that PANI and CNT@BaTiO_3_ displayed a relatively poor microwave absorption performance, and the minimum reflection loss of PANI and CNT@BaTiO_3_ reached −11.3 dB at 7.0 GHz/8.1 GHz and −11.2 dB at 15 GHz, respectively. The obtained CNT@BaTiO_3_@PANI exhibited a breakthrough in the improvement of microwave absorption. It shows that the minimum reflection loss reached −17.8 dB (absorbing more than 98%) at 15 GHz and the frequency bandwidth corresponding to the reflection loss at −10 dB was 3 GHz (from 12 to 15 GHz). The superior performance of the CNT@BaTiO_3_@PANI composites may be caused by the balance of dielectric loss and magnetic loss, inducing a better impedance match [40].

**Figure 8:**
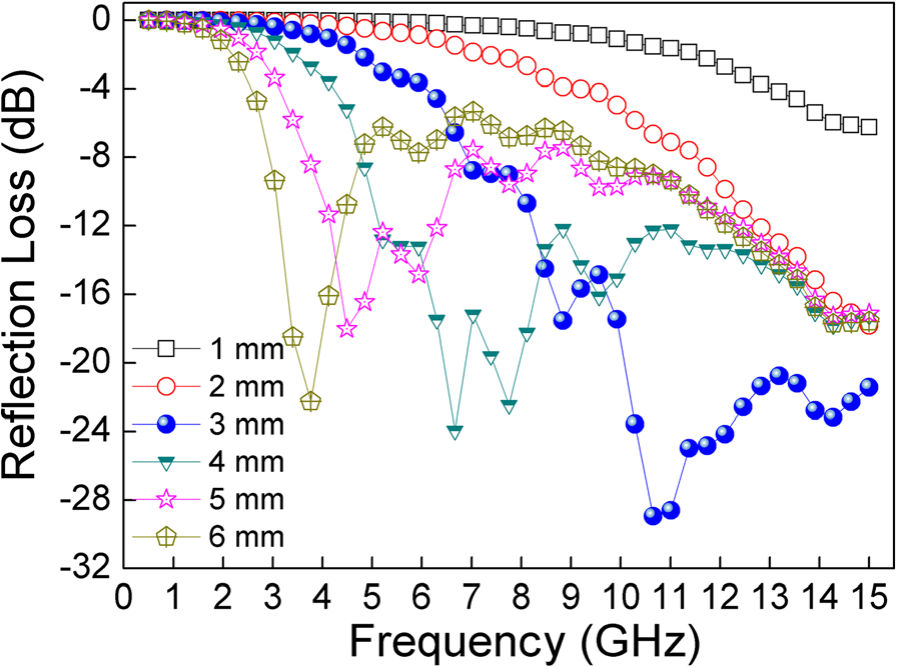
Thickness dependence of reflection loss for CNT@BaTiO_3_@PANI composite with 20 wt % loadings in paraffin wax. In the frequency range 0.5 to 15 GHz.

Figure 8 shows the reflection loss of mixtures of CNT@BaTiO_3_@PANI composites with 20 wt % loadings in paraffin wax coupled with different thicknesses. The microwave absorbing parameters for the samples are listed in Table 1. It can be observed that the reflection loss peaks shift from a higher to a lower frequency while the thickness increased, associated with quarter-wavelength attenuation. The minimum RL reached −28.9 dB (approximately 99.87% absorption) at 10.7 GHz with a thickness of 3 mm, and the frequency bandwidth was less than −20 dB from 10 to 15 GHz. Moreover, it could be observed that the minimum RL values were less than −17.8 dB when the thickness was in the range of 2 ~ 6 mm. The excellent microwave absorption of the CNT@BaTiO_3_@PANI composites might be attributed to the better impedance match. It was also believed that the special multiphase heterostructure of the CNT@BaTiO_3_@PANI composites can produce an expanded propagation path of electromagnetic wave through multiple reflections leading to an excellent broadband microwave absorbing performance. Since microwave absorbers with reflection loss values less than −10 dB can be designed to attenuate electromagnetic waves, the one-dimensional CNT@BaTiO_3_@PANI multiphase heterostructure composites are very promising lightweight electromagnetic wave absorption materials.

**Table 1:** **Microwave absorption properties of samples**

**Sample (thickness of CNT@BaTiO**_**3**_ **@PANI)**	**Microwave absorption properties of samples**			
	**RL**_**m**_ **(dB)**	***f***_**m**_ **(GHz)**	**Frequency range (GHz) (RL < −10 dB)**	**Frequency range (GHz) (RL < −20 dB)**
1 mm	−6.25	15.0	0	0
2 mm	−17.82	15.0	12.1 ~ 15	0
3 mm	−28.95	10.7	7.9 ~ 15	10 ~ 15
4 mm	−23.94	6.7	5 ~ 15	6.4 ~ 6.9/7.5 ~ 8
5 mm	−18.01	4.5	4.0 ~ 6.5/11.4 ~ 15	0
6 mm	−22.27	3.7	3.1 ~ 4.6/11.4 ~ 15	3.5 ~ 3.9

RL_m_, minimum reflection loss value; *f*_m_, frequency at which the reflection loss is at its minimum.

## Conclusions

In conclusion, a one-dimensional CNT@BaTiO_3_@PANI multiphase heterostructure composite was successfully fabricated via coupled sol–gel method and *in situ* polymerization. The structures, morphology, conductive properties, and microwave absorption performance of the composites have been characterized. The CNT@BaTiO_3_@PANI composites showed the best reflection loss of −28.9 dB at 10.7 GHz with a thickness of 3 mm, and a frequency bandwidth less than −20 dB was observed from 10 to 15 GHz. The excellent microwave absorption property was due to their special structural characteristics, well-matched characteristic impedances, and interfacial polarization induced by multiple interfaces in the composites. Furthermore, the content of the CNT@BaTiO_3_@PANI composites in the paraffin matrix was only 20 wt %, much lower than that of others recently reported. We believe that the one-dimensional CNT@BaTiO_3_@PANI heterostructures are very promising as effective lightweight fillers in highly effective electromagnetic attenuation.
